# Electronic screening for mental illness in patients with psoriasis

**DOI:** 10.1093/bjd/ljad141

**Published:** 2023-05-24

**Authors:** Katie Bechman, Joseph F Hayes, Julian Mathewman, Alasdair D Henderson, Elizabeth I Adesanya, Kathryn E Mansfield, Catherine H Smith, James Galloway, Sinéad M Langan

**Affiliations:** Centre for Rheumatic Diseases, King’s College London, London, UK; Division of Psychiatry, University College London, London, UK; Department of Non-communicable Disease Epidemiology, London School of Hygiene & Tropical Medicine, London, UK; Department of Non-communicable Disease Epidemiology, London School of Hygiene & Tropical Medicine, London, UK; Department of Non-communicable Disease Epidemiology, London School of Hygiene & Tropical Medicine, London, UK; Department of Non-communicable Disease Epidemiology, London School of Hygiene & Tropical Medicine, London, UK; St John’s Institute of Dermatology, NIHR Biomedical Research Centre, Guy's and St Thomas' NHS Foundation Trust, King’s College London, London, UK; Centre for Rheumatic Diseases, King’s College London, London, UK; Department of Non-communicable Disease Epidemiology, London School of Hygiene & Tropical Medicine, London, UK

## Abstract

In this cross-sectional study from a large UK centre, screening for mental illness in individuals with psoriasis has demonstrated a high burden of depression and anxiety. Overall, 85% of the cohort reported that their psoriasis had affected their quality of life. Quality-of-life scores correlate with depression scores, emphasizing the importance of managing the individual’s mental health alongside their psoriasis to improve overall quality of life.

https://doi.org/10.1093/bjd/ljad141

Dear Editor, Individuals with psoriasis have an increased risk of depression, anxiety and severe mental illness.^1,2^ Guidelines provided by the National Institute for Health and Care Excellence recommend assessing for mental health alongside psoriasis disease severity and disease impact. Systematic screening for depression and anxiety symptoms in tertiary centres has identified a significant burden of disease^3^ and has led to increased use of mental healthcare and improvement in psoriasis and quality of life.^1,4^

This cross-sectional study examined the use of screening for mental illness in a large centre serving London and Southeast England. Individuals with a confirmed psoriasis diagnosis attending Guy’s and St Thomas’ National Health Service (NHS) Foundation Trust and King’s College Hospital, London (from January 2017 to January 2020) were invited to answer a series of questions about their health at every outpatient visit. A touchscreen tablet-based programme, Integrating Mental and Physical Healthcare: Research Training and Services (IMPARTS) was used to collect patient-completed screening questionnaires including the Patient Health Questionnaire (PHQ-9),^5^ Generalized Anxiety Disorder (GAD-7) scale^6^ and the Dermatology Life Quality Index (DLQI).^7^ IMPARTS is a multifaceted platform of clinical and research services that integrates mental healthcare into routine care.^3^ Completed questionnaire data automatically populates the patient’s electronic health record with advice on mental health referral if questionnaire scores suggest a possible mental health condition. We performed statistical analyses using Stata (StataCorp, College Station, TX, USA). We assessed cross-sectional correlations between screening questionnaires using Spearman’s correlation coefficient. We used linear regression, adjusting for age, sex and year of visit and clustering for repeat questionnaires by individual patients to examine the relationship between DLQI and mental health.

All data used in this study are held on a restricted server at King’s College Hospital NHS Foundation Trust behind the Trust firewall and in line with Trust policies, as is the case with all other clinical data. Data held on the IMPARTS server are accessible only to staff members working on the IMPARTS programme, and data processors by approval. Information relevant to patient care is added to the patient’s electronic care record. Data may be used to monitor the delivery of the IMPARTS programme, for clinical audit, and service evaluation. Aggregated IMPARTS data may be used to publish research at various levels. IMPARTS programme ethical approval was provided for this study (IMPARTS Research Database Research Ethics Committee reference: 12/SC/0422). All applications to use data routinely collected under the IMPARTS programme are scrutinized by a patient-led oversight committee to ensure that the use of data is appropriate and in line with ethics committee approval.

Engagement in screening for mental illness rose gradually over time with substantial month-to-month variation (Figure 1a). In total, 285 individuals provided data. Of these, 217 individuals provided data at more than one visit [the median number of visits was three, interquartile range (IQR) 2–4 over a median time of 1.5 years (IQR 0.9–2)]. The median age was 42 years (IQR 31–53) and there was a slight male predominance (*n* = 147, 52%) in our study cohort. On the first recorded visit, one-third of the cohort screened positive for psoriatic arthritis (Psoriasis Epidemiology Screening Tool).

**Figure 1:**
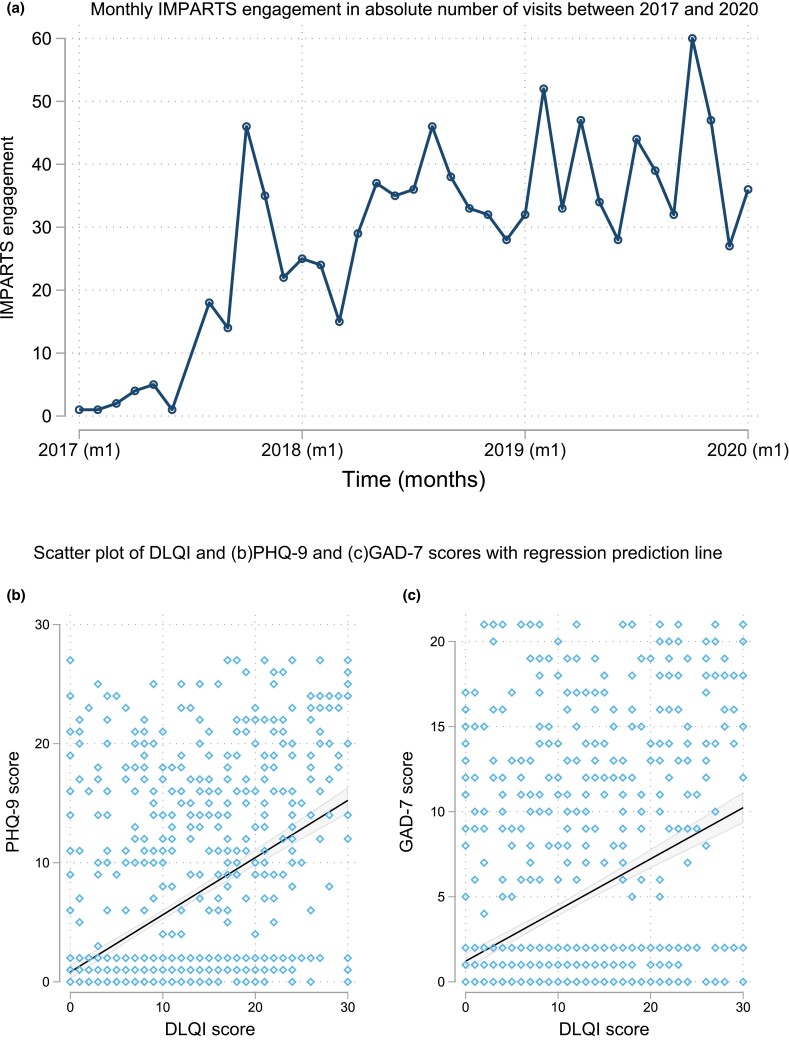
(a) Monthly Integrating Mental and Physical Healthcare: Research Training and Services (IMPARTS) engagement provided as absolute number of visits between 2017 and 2020. (b) Scatter plot of Dermatology Life Quality Index (DLQI) and Patient Health Questionnaire (PHQ-9) score with regression prediction line. (c) Scatter plot of DLQI and Generalized Anxiety Disorder (GAD-7) scores with regression prediction line.

At first visit, 84% (*n* = 238) of the cohort reported that their psoriasis had affected their quality of life (DLQI score > 2). Quality-of-life impairment was reported as very large (DLQI score 11–20) by 32% (*n* = 90) or extremely large (DLQI score 21–30) by 17% (*n* = 47). The depression screen, defined as a positive answer to either of the first PHQ-9 questions, was present in 35% (*n* = 100). The anxiety screen, defined by a GAD-7 score > 5 was positive in 29% (*n* = 82). Severe depressive symptoms (PHQ-9 ≥ 20) were reported by 22% (*n* = 60) and severe anxiety symptoms (GAD-7 ≥ 15) were reported by 23% (*n* = 64).

When examining across all visits, quality-of-life impairment was frequently reported (at 78% of visits patients had a DLQI score > 2). The median DLQI score was 8 (IQR 2–16). Depression screen was positive on 30% of visits and anxiety on 25% of visits. The median PHQ-9 score was 1 (0–5) and the median GAD-7 score was 2 (0–10). Severe depression and anxiety symptoms were more frequently reported by women than men (PHQ-9, women 24% vs. 15% men; GAD-7, 25% vs. 14%), as was severe quality-of-life impairment (47% vs. 33%).

Across all visits, the DLQI score moderately correlated with the PHQ-9 score (*ρ* = 0.52) (Figure 1b) and weakly correlated with the GAD-7 scores (*ρ* = 0.41) (Figure 1c). In linear regression, depression and anxiety were associated with DLQI scores [PHQ-9: *β* = 0.48, 95% confidence interval (CI) 0.38–0.56, *P* < 0.001; GAD7: *β* = 0.29, 95% CI 0.21–0.36, *P* < 0.001]. For each one-unit increase in PHQ-9, DLQI score increased by half a point (*r*^2^ = 0.27), and for each one-unit increase in GAD-7, DLQI increased by one-third of a point (*r*^2^ = 0.18).

Our study demonstrated increasing engagement in the screening of psychological wellbeing over time, and the burden of depression and anxiety in people with psoriasis. We also demonstrated a strong relationship between mental health and quality of life. Our findings emphasize the importance of holistic care and managing the individual’s mental health alongside their psoriasis to improve overall quality of life.
